# Editorial: advances in deep learning techniques for biomedical imaging

**DOI:** 10.1186/s42492-023-00139-2

**Published:** 2023-06-21

**Authors:** Chuang Niu, Ge Wang

**Affiliations:** Department of Biomedical Engineering, School of Engineering, Biomedical Imaging Center, Center for Biotechnology and Interdisciplinary Studies, Rensselaer Polytechnic Institute, Troy, NY 12180 USA

The field of biomedical imaging has been revolutionized by deep learning techniques. This special issue is focused on the theme of “AI-based Image Analysis”. Because there are so many conferences and journals in this field, our special issue can only be a small snapshot of a much bigger and highly dynamic picture.

In this special issue, we present six papers that highlight the power of deep learning in solving challenging biomedical imaging and image analysis problems.

In the first paper, Qi and Wang demonstrate the superiority of quadratic neural networks over conventional neural networks in the context of classifying Gaussian mixture data, where both the theoretical upper bound and classic EM algorithms are well established. They systematically compare quadratic and conventional neural networks for classification, and show that quadratic neural networks are clearly more efficient and effective than conventional neural networks.

The second paper by Fu and De Man presents a novel framework for deep tomographic reconstruction through hierarchical domain decomposition. They cast the original problem as a continuum of intermediate representations between the input and output domains and break down the problem into a sequence of simpler transformations that can be mapped onto an efficient network architecture. The proposed approach is demonstrated on full-scale CT image reconstruction.

In the third paper, Wang, Li, and Haverinen present a novel approach for real-time photon-counting CT-based thermometry through material decomposition and machine learning. Traditional CT thermometry is based on energy-integrated detectors, tissue-specific experimental data, and linear relationships between x-ray attenuation and temperature. This paper introduces a new method that can handle non-linear thermal properties of composite materials. The experimental results show that the neural network trained on the photon-counting spectral tomographic measurements produce promising results. This approach has a great potential in thermotherapies such as high-intensity focused ultrasound enabled thermal ablation of tumors.

In the fourth paper, Pack et al. review cardiac CT blooming artifacts, their clinical significance, root causes, and potential solutions. They study more than 30 key journal papers and identify the partial volume, motion blurring, and beam hardening as the main components of blooming artifacts. They propose solutions including high-quality CT hardware, high-resolution CT reconstruction, and post-processing techniques, with a special emphasis on deep learning techniques.

In the fifth paper, Yang et al. make a preliminary landscape analysis of deep tomographic imaging patents. With the increasing importance of the patent literature in the academic and industrial settings, they analyze the related patent literature for deep tomographic imaging. PatSeer is used as a search tool to extract the elevant patent bibliometric data. The qualitative analysis of the key deep tomographic patents offers insights into the field and its potential.

Finally, Zhang presents a machine learning method for enumeration of cell colony forming units. The method, called CFUCounter, combines unsupervised machine learning, iterative adaptive thresholding, and local-minima-based watershed segmentation for accurate and robust cell counting. The results show that CFUCounter performs as accurately as the gold standard of point-and-click counting. Clearly, CFUCounter is a unique addition to the arsenal of colony-counting tools.

As the special issue editors, we are happy with the decent quality of all the papers and appreciate outstanding review reports prepared by reviewers. Indeed, all these papers showcase the potential of deep learning techniques in solving biomedical imaging problems, ranging from image classification, reconstruction and artifact correction to cell counting. We hope that this special issue will inspire further research in this field and lead to more breakthroughs in the future.

